# Clinical outcome comparison of laparoscopic radical antegrade modular pancreatosplenectomy vs. laparoscopic distal pancreatosplenectomy for left-sided pancreatic ductal adenocarcinoma surgical resection

**DOI:** 10.3389/fsurg.2022.981591

**Published:** 2022-09-01

**Authors:** Nan Niu, Yuhui He, Yiping Mou, Sijia Meng, Peng Xu, Yucheng Zhou, Weiwei Jin, Chao Lu, Yunyun Xu, Qicong Zhu, Tao Xia

**Affiliations:** ^1^ Department of Surgery, The Second Clinical Medical College of Zhejiang Chinese Medical University, Hangzhou, China; ^2^ Department of General Surgery, Cancer Center, Division of Gastrointestinal and Pancreatic Surgery, Zhejiang Provincial People’s Hospital, Affiliated People’s Hospital, Hangzhou Medical College, Hangzhou, China; ^3^ Department of Surgery, Bengbu Medical College, Bengbu, China; ^4^ Department of Surgery, Qingdao University, Qingdao, China

**Keywords:** pancreatic cancer, laparoscopic distal pancreatosplenectomy, laparoscopic RAMPS, chemotherapy, survivability

## Abstract

**Background:**

Laparoscopic radical antegrade modular pancreatosplenectomy (LRAMPS) is a validated surgical treatment for patients with left-sided pancreatic ductal adenocarcinoma (PDAC). In addition, laparoscopic distal pancreatectomy (LDPS) has purported benefits. However, there is a limited analysis comparing the results between LRAMPS and LDPS. Thus, this study aims to compare the short-term and long-term outcomes of patients who underwent LRAMPS and LDPS for PDAC treatment.

**Methods:**

Patients with left-sided PDAC that underwent LRAMPS or LDPS from 2015 to 2021 were retrospectively identified. Demographic and clinic pathologic data were collected. Disease-free survival (DFS) and overall survival (OS) probabilities were obtained.

**Results:**

The number of lymph nodes retrieved was significantly greater in the LRAMPS group than in the LDPS group. Several clinicopathological factors, including CA19-9 levels greater than 37 U/ml, positive lymph nodes, moderate to poor tumor differentiation, and peripancreas fat invasion, were associated with DFS. Moderate with poor tumor differentiation was associated with poor DFS (HR 0.568; 95% CI 0.373–0.921; *P* = 0.021). Levels of CA19-9 greater than 37 U/ml, CEA levels greater than 5 μg/ml, larger tumor size, positive lymph nodes, moderate with poor tumor differentiation, peripancreas fat invasion, and adjuvant chemotherapy were all associated with OS. LRAMPS nearly improved OS but did not reach statistical significance. Serum carcinoembryonic antigen (CEA) levels greater than 5 ug/ml (HR 1.693; 95% CI 1.200–1.132; *P* = 0.001), and positive lymph nodes (HR 2.410; 95% CI 1.453–3.995; *P* = 0.001) were independently associated with poor OS. Treatment with adjuvant chemotherapy was associated with improved OS (HR 0.491; 95% CI 0.248–0.708; *P* = 0.001).

**Conclusions:**

The LRAMPS procedure achieved comparable results to standard LDPS in terms of postoperative outcomes. Treatment with chemotherapy is important for the prognosis of patients with left-sided pancreatic cancer.

## Introduction

Surgical resection offers the only curative treatment for pancreatic ductal adenocarcinoma (PDAC). Specifically, distal pancreatectomy (DPS) is the standard procedure for left-sided PDAC resection, but it has a high rate of retroperitoneal margin positivity (62%), which indicates that cancerous tissue persists post-surgery. In 2003, Dr. Steven Strasberg introduced the radical antegrade modular pancreatosplenectomy (RAMPS) procedure (1). Compared to DPS, RAMPS attempts to achieve negative retroperitoneal margins and higher lymph node retrieval in order to improve survival outcomes (2, 3). Several studies have demonstrated that RAMPS increases the negative tangential margin rate and lymph node harvest (4). Furthermore, laparoscopic distal pancreatectomy (LDPS) has purported benefits for short-term and long-term outcomes compared to open DPS (5, 6). Similarly, laparoscopic RAMPS (LRAMPS) has been demonstrated to be more feasible (7–9). In a recent meta-analysis to date comparing minimally invasive RAMPS (MI-RAMPS) against open RAMPS, intra-operative blood loss was observed to be significantly reduced in MI-RAMPS, while lymph node yield was higher in O-RAMPS and there was no difference in overall survival (OS) (10). Modern chemotherapy has proven to be effective and improves the survival of PDAC. A previous study revealed chemotherapy to be an independent factor for OS after RAMPS and DPS (11). However, there is limited clinical outcome analysis comparing the LRAMPS and LDPS procedures with chemotherapy. Thus, we conducted this study to compare the short-term and long-term outcomes of patients who underwent LRAMPS and LDPS for left-sided PDAC treatment.

## Methods

### Patients

From May 2015 to May 2021, patients with left-sided PDAC that underwent LRAMPS or LDPS procedures performed by one surgeon were retrospectively identified from the database at Zhejiang Provincial People's Hospital. Patients with left-sided PDAC without any evidence of distant metastasis or vascular invasion beyond the celiac axis were included. Patients who underwent neoadjuvant therapy and vascular reconstruction were excluded. All acquisition methods were approved by the Institutional Review Board.

Demographic and clinic pathologic data were collected. Postoperative pancreatic fistula (POPF) was defined according to the International Study Group on Pancreatic Fistula Definition (ISGPF) (12). All patients with grades B and C were defined as having clinically significant POPF. The severity of complications was defined according to Clavien–Dindo classification system (13). Pathological data were classified using the eighth AJCC/UICC TNM. R1 margin status was defined as <1 mm from the edge of the specimen. Overall survival (OS) was defined as the duration of time between the date of surgery and the date of death. Disease-free survival (DFS) was similarly calculated as the interval between the date of surgery and the date of tumor recurrence. Adjuvant chemotherapy was recommended as routine therapy for patients who could tolerate the adverse effects. Chemotherapy regimens and duration were left to the discretion of the oncologist.

#### Operative procedures

The LRAMPS procedure was performed in our institution since 2017 as described by Strasberg et al. (1). Briefly, patients were placed in the supine position with their heads slightly elevated. Five trocars, including a camera port (10 mm) below the umbilicus and four additional working ports (one 12 mm and three 5 mm), were placed in the right flank, right upper flank, left upper flank, and left flank. The peritoneal cavity and liver surface were inspected to rule out metastasis. The gastrocolic ligament was divided to expose the anterior surface of the pancreas. The inferior border of the pancreas was mobilized to visualize the superior mesenteric vein and the portal vein. Furthermore, the pancreas was mobilized along the superior border to explore the common hepatic artery and to dissect the lymph nodes along the common hepatic and gastroduodenal arteries. The pancreas neck was then transected with an endoscopic linear stapler (Ethicon Endo-Surgery, PSE45A, Cincinnati, OH, USA). The splenic artery and vein were ligated and divided, respectively. Lymph nodes were dissected from the celiac axis down to the left side of the superior mesenteric artery. The distal pancreas was dissected along with the soft tissue of the retroperitoneum in a medial-to-lateral manner. The short gastric vessels were ligated to mobilize the spleen and the lymph nodes along the splenic artery and hilum were removed. After complete resection of the distal pancreas and spleen, the tissue was removed through an enlarged umbilical incision with a specimen bag. The LDPS procedure was performed as previously described (14). The laparoscopic procedure was performed as the LRAMPS procedure. The lymph nodes along the celiac axis and superior mesenteric artery were dissected but the soft tissue of the retroperitoneum was reserved.

#### Statistical analysis

All statistical analyses were performed using SPSS v.22.0 and GraphPad Prism 8 software. All continuous variables were presented as median summative values with interquartile ranges (IQRs). Continuous variables were compared using the student's *t*-test or Wilcoxon rank test for parametric or nonparametric distributions, respectively. The chi-square or Fisher's exact tests were used to analyze categorical variables, as appropriate. OS and DFS were assessed using the Kaplan–Meier estimate method, and comparisons were conducted using the log-rank test. Only variables with *P*-value less than 0.05 in univariate analysis were included in a Cox proportional hazards model. *P*-values less than 0.05 were considered statistically significant.

## Results

In total, 109 patients with left-sided PDAC underwent surgical resection. Clinic pathological characteristics of patients are summarized in Table 1.

**Table 1:** Clinicopathological characteristics of patients with left-side pancreatic ductal adenocarcinoma.

Variables	All cases (*N* = 109)	LRAMPS (*N* = 50)	LDPS (*N* = 59)	*P* value
Sex, female	41 (37.6%)	19 (38%)	22 (37.3%)	0.939
Age (years)	67 [38,88]	67 [44,87]	66 [38,88]	0.588
BMI (kg/m^2^)	22.37 [14.76,30.47]	22.30 [17.58,30.09]	22.39 [14.76,30.47]	0.081
CA19-9 (>37 U/L)	83 (76.1%)	37 (74%)	46 (78%)	0.628
CEA (>5 U/L)	39 (35.8%)	18 (36%)	21 (35.6%)	0.965
Estimated blood loss (ml)	100 [40,300]	100 [50,300]	100 [40,200]	0.627
Operative time (min)	130 [90,310]	140 [100,310]	130 [90,230]	0.351
Pancreatic fistula, B and C	19 (17.4%)	6 (12%)	13 (22%)	0.169
Clavien–Dindo, I/II, III	98/11	46/4	52/7	0.505
Length of stay (days)	12 [7,56]	13 [7,56]	12[7,44]	0.940
Tumor size (cm)	3.5 [1.5,10]	3.5 [1.8,10]	3.5 [1.5–7.0]	0.799
Retrieved lymph nodes	9 [5,28]	11 [5,28]	7 [5–28]	0.035
Resection margin status	5 (9.4%)	3 (6%)	2 (3.4%)	0.659
Differentiation, well-moderate/moderate with poor	59/50	25/25	34/25	0.426
Perineural invasion, yes/no	86/23	44/6	42/17	0.056
Vascular invasion, yes/no	37/72	19/31	18/41	0.401
Peripancreas fat invasion, yes/no	77/32	34/16	43/16	0.577
Adjuvant chemotherapy, yes/no	48/61	23/27	25/34	0.704

LRAMPS, laparoscopic radical antegrade modular pancreatosplenectomy; LDPS, laparoscopic distal pancreatectomy; CA, carbohydrate antigen; CEA, carcinoembryonic antigen.

Fifty (45.9%) patients underwent LRAMPS, and 59 (54.1%) patients underwent LDPS. Factors including sex, age, BMI, serum carbohydrate antigen (CA) 19-9 values, and serum carcinoembryonic antigen (CEA) values were confirmed to have no significant difference between the LRAMPS and LDPS groups. A total of 19 patients developed clinically significant POPF. Thirteen cases of grade B POPF occurred in the LDPS group. Moreover, one case of grade C and five cases of grade B POPF were confirmed in the LRAMPS group. No meaningful differences were found in operative time, estimated blood loss (EBL), clinically significant POPF, complications, or length of stay (Table 1).

There were no significant differences in the tumor size, level of differentiation, R0 resection margin, perineural invasion, vascular invasion, or peripancreas fat invasion. The number of lymph nodes retrieved was significantly greater in the LRAMPS group than in the LDPS group (*P* = 0.035). Furthermore, 49 (42.2%) patients received adjuvant chemotherapy and others rejected chemotherapy. Gemcitabine-based chemotherapy was administered as first-line chemotherapy for most patients; the remaining patients received FOLFIRINOX. The median follow-up period was 46.7 months.

By the end of the follow-up period, disease recurrence occurred in 77 (70.64%) patients and 69 (63.3%) patients had died. The median DFS was 12.6 months and the median OS was 22.9 months. Finally, the 1-year, 3-year, and 5-year OS rates were 84.4%, 25.7%, and 12.8%, respectively.

Several clinicopathological factors were associated with DFS upon univariate analysis, including CA19-9 levels greater than 37 U/ml, positive lymph nodes, moderate with poor tumor differentiation, and peripancreas fat invasion. Neither surgical procedure nor adjuvant chemotherapy was found to affect DFS. Additionally, moderate with poor tumor differentiation was associated with poor DFS upon multivariate analysis (HR 0.568; 95% CI 0.373–0.921; *P* = 0.021) (Table 2).

**Table 2:** Kaplan–Meier and Cox proportional hazard regression analysis of disease-free survival.

Variables	Univariate		Multivariate		
	Median survival (months)	*P* value	HR	95% CI	*P* value
Sex (male/female)	11.00/17.40	0.401			
Age (≤74/≥75 years)	12.60/12.10	0.601			
BMI (≤18.4/≥18.5 kg/m^2^)	9.60/12.70	0.464			
CA19-9 (<37/≥37 U/L)	41.30/11.80	0.017	1.757	0.970–1.259	0.063
CEA (<5/≥5 U/L)	13.20/11.00	0.098			
LRAMPS/LDPS	11.80/14.70	0.544			
T (T1/T2, T3)	54.90/12.40	0.193			
Positive lymph nodes (yes/no)	9.70/15.60	0.003	1.352	0.793–2.303	0.268
Resection margin status (R0/R1)	12.60/12.10	0.922			
Differentiation (moderate with poor/well-moderate)	9.20/17.30	0.010	0.568	0.373–0.921	0.021
Perineural invasion (yes/no)	12.40/12.60	0.641			
Lymphatic invasion (yes/no)	16.20/12.10	0.757			
Peripancreas fat invasion (yes/no)	12.00/13.00	0.020	0.598	0.341–1.050	0.074
Adjuvant chemotherapy (yes/no)	17.40/11.8	0.095			

LRAMPS, laparoscopic radical antegrade modular pancreatosplenectomy; LDPS, laparoscopic distal pancreatectomy; CA, carbohydrate antigen; CEA, carcinoembryonic antigen.

Univariate analysis revealed CA19-9 levels greater than 37 U/ml, CEA levels greater than 5 ug/ml, larger tumor size, positive lymph nodes, moderate with poor tumor differentiation, peripancreas fat invasion, and adjuvant chemotherapy were associated with OS. LRAMPS trended to improve OS but did not reach statistical significance (28.83 months vs. 18.93 months, *P* = 0.336). Serum CEA levels greater than 5 μg/ml (HR 1.693; 95% CI 1.200–1.132; *P* = 0.001) and positive lymph nodes (HR 2.410; 95% CI 1.453–3.995; *P* = 0.001) were independently associated with poor OS. Treatment with adjuvant chemotherapy was associated with improved OS (HR 0.491; 95% CI 0.248–0.708; *P* = 0.001) (Table 3).

**Table 3:** Kaplan–Meier and Cox proportional hazard regression analysis of overall survival.

Variables	Univariate		Multivariate		
	Median survival (months)	*P* value	HR	95% CI	*P* value
Sex (male/female)	20.00/28.83	0.425			
Age (≤74/≥75 years)	25.80/16.50	0.108			
BMI (≤18.4/≥18.5 kg/m^2^)	21.80/24.27	0.697			
CA19-9 (<37/≥37 U/L)	46.33/20.00	0.019	1.680	0.893–3.161	0.107
CEA (<5/≥5 U/L)	26.43/16.60	0.010	1.963	1.200–3.212	0.007
LRAMPS/LDPS	18.93/28.83	0.336			
T (T1/T2, T3)	No/22.93	0.126			
Positive lymph nodes (yes/no)	16.80/29.53	0.002	2.410	1.453–3.995	0.001
Resection margin status (R0/R1)	22.93/33.27	0.822			
Differentiation (moderate with poor/well-moderate)	17.60/33.53	0.017	0.661	0.386–1.132	0.132
Perineural invasion (yes/no)	23.33/22.93	0.959			
Lymphatic invasion (yes/no)	22.93/23.33	0.642			
Peripancreas fat invasion (yes/no)	21.53/29.77	0.034	0.762	0.404–1.437	0.401
Adjuvant chemotherapy (yes/no)	33.53/16.50	0.001	0.419	0.248–0.708	0.001

LRAMPS, laparoscopic radical antegrade modular pancreatosplenectomy; LDPS, laparoscopic distal pancreatectomy; CA, carbohydrate antigen; CEA, carcinoembryonic antigen.

**Figure 1:**
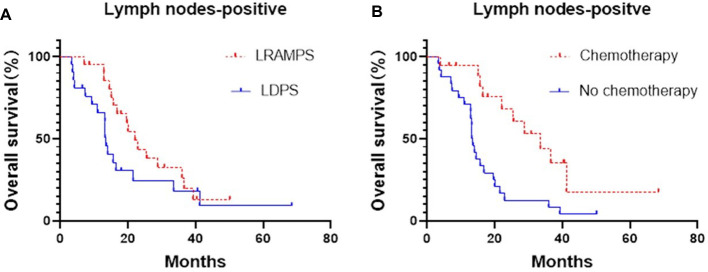
Median OS of positive lymph nodes patients, (**A**) LRAMPS: 22.1 months; LDPS: 13.57 months. (**B**) Chemotherapy: 33.5 months; no chemotherapy: 13.4 months.

**Figure 2:**
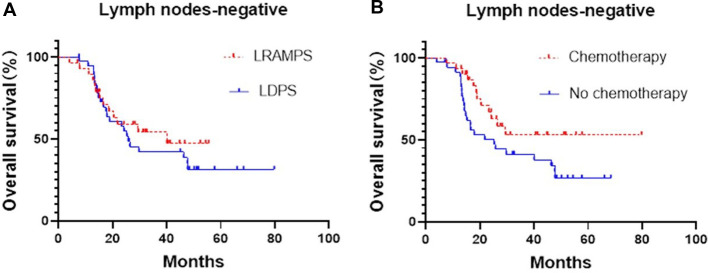
Median OS of negative lymph nodes patients, (**A**) LRAMPS: 40.1 months; LDPS: 25.8 months. (**B**) Chemotherapy: not reach; no chemotherapy: 25.3 months.

Cancer-positive lymph nodes were found to affect both DFS and OS. The LRAMPS procedure retrieved more lymph nodes than LDPS. However, the median OS of patients with positive lymph nodes that underwent LRAMPS and LDPS was 22.1 and 13.6 months, respectively, and did not reach statistical significance (*P* = 0.156). OS in patients with positive lymph nodes and treated with adjuvant chemotherapy was significantly different from untreated patients (33.5 months vs. 13.4 months, *P* = 0.001) (Figure 1). In the cancer-negative lymph node cohort, neither surgical procedure (*P* = 0.502) nor treatment with adjuvant chemotherapy (*P* = 0.065) was found to affect OS (Figure 2).

## Discussion

Curative surgery plays an essential role in the treatment of PDAC. R0 resection margin and radical N1 lymph node resection are major prognostic factors for patients with left-sided PDAC (15, 16). Many reports (11, 17) showed that RAMPS achieved improved R0 resection and regional lymph node retrieval compared to DPS as evident by reported outcomes. Due to these results, RAMPS became a mainstream surgical procedure for patients with left-sided PDAC. Over the past decade, the safety and feasibility of LDPS improved and demonstrated fewer complications with shortened hospital stays worldwide (5). Therefore, the LRAMPS procedure was mainly performed in high-volume pancreatic centers, where several reports indicated the improved safety and feasibility of LRAMPS (10).

The LRAMPS procedure is more complex than LDPS. Therefore, surgeons worried about longer operative times and perioperative complications when deciding between the two procedures. However, in the present study, there were no significant differences found in operative time, EBL, clinically significant POPF, complications, and length of stay between LRAMPS and LDPS groups. A large, multi-institutional study also revealed that there were no differences in rates of postoperative pancreatic fistula (16.5% vs. 17.8%, *P* = 1.000) and postoperative hemorrhage (5.9% vs. 3.6%, *P* = 0.385) between RAMPS and DPS groups (18). Furthermore, a meta-analysis indicated no significant differences were observed between RAMPS and DPS in terms of postoperative complications (*P* = 0.87), POPF (*P* = 0.15), mortality (*P* = 0.80), and length of stay (*P* = 0.53) (4).

This study demonstrated no significant difference in DFS or OS between LRAMPS and LDPS procedures and supported the findings of previous studies and meta-analyses of RAMPS and DPS (11, 18). Moreover, the results showed that adjuvant chemotherapy was associated with improved OS. It supported the consensus that adjuvant chemotherapy is indispensable for patients with PDAC. Survival is slowly increasing with a reported median OS of 54.4 months with adjuvant chemotherapy with FOLFIRINOX after surgery (19, 20).

Many RAMPS proponents argued that R0 resection and more lymph node retrieval can improve prognosis. In this study, the number of lymph nodes retrieved was significantly greater in the LRAMPS group than in the LDPS group. However, there was no significant difference in the survival of patients with positive lymph nodes that underwent LRAMPS or LDPS (22.1 months vs. 13.6 months, *P* = 0.156). In addition, we found no significant difference in the R0 resection margin. Moreover, OS in patients with positive lymph nodes and treated with adjuvant chemotherapy was significantly longer than in patients that did not receive adjuvant chemotherapy (33.5 vs. 13.4, *P* = 0.001). It was consistent with several reports between open RAMPS and DPS (11, 18). We hypothesize the survival of patients with positive lymph nodes was more likely determined by adjuvant chemotherapy because of biological properties rather than surgical procedures (21). Modern chemotherapy has proven to be effective and improve survival, even in the setting of an R1 resection (22, 23).

We acknowledge that this study has limitations. First, this was a retrospective, cohort study, which has inherent weaknesses. Second, some data, such as pancreatic parenchyma texture and the thickness of the pancreatic stump, were not elaborately collected and may affect the quality of the reported short-term outcomes. Finally, due to the limited number of patients who received adjuvant chemotherapy, the efficacy of different chemotherapy regimens could not be thoroughly evaluated.

## Conclusions

The LRAMPS procedure achieves comparable results to standard LDPS regarding postoperative outcomes. Comparatively, LRAMPS retrieves more lymph nodes. In addition, positive lymph nodes correlated with both DFS and OS. However, LRAMPS is not associated with an improvement in either DFS or OS over LDPS. OS in patients with positive lymph nodes and treated with adjuvant chemotherapy was significantly better than in untreated patients. Treatment with chemotherapy is an important predictive factor procedure for the prognosis of patients with left-sided pancreatic cancer.

## Data Availability

The original contributions presented in the study are included in the article/Supplementary Material, further inquiries can be directed to the corresponding author/s.
